# A critical analysis of health system in Nepal: Perspectives based on COVID-19 response

**DOI:** 10.1016/j.dialog.2023.100142

**Published:** 2023-06-10

**Authors:** Bihari Sharan Kuikel, Archana Shrestha, Dong Roman Xu, Brish Bahadur Shahi, Bakhat Bhandari, Ravi Kanta Mishra, Navaraj Bhattrai, Kiran Acharya, Ashish Timalsina, Nripa Raj Dangaura, Bikram Adhikari, Rabin Dhital, Biraj Man Karmacharya

**Affiliations:** a Department of Public Health and Community Programs, Kathmandu University School of Medical Sciences, Dhulikhel, Nepal; b Institute for Implementation Science and Health, Kathmandu, Nepal; c Center of Methods for Implementation and Prevention Science, Yale School of Public Health, New Haven, CT, USA; d Southern Medical University, Guangzhou, China; e Ministry of Social Development, Karnali Province, Nepal; f Ministry of Health and Population, Kathmandu, Nepal; g Nepal Public Health Foundation, Kathmandu, Nepal; h New ERA, Kathmandu, Nepal; i Family Welfare Division, Department of Health Services, Kathmandu, Nepal; j HERD International, Lalitpur, Nepal; k Rural Development and Empowerment Center, Nuwakot, Nepal

**Keywords:** WHO health system building blocks, COVID-19, Health system, Nepal

## Abstract

**Background:**

Nepal moved from a unitary government to a federal system of government in 2015 under its constitution. Nepal is a federal democratic republic governed by three levels of government: a federal, provincial, and local level. The response to COVID-19 in Nepal has been majorly led and controlled by the federal government. All three levels of government are performing their responsibilities; however, they face various challenges in responding to COVID-19. This study aimed to critically analyze Nepal's health system in the context of the COVID-19 response.

**Methods:**

We conducted semi-structured in-depth interviews by telephone among the policymakers, health workers, and stakeholders at the federal, provincial, and local levels (*n* = 41) between January to July 2021. The interviews were audio recorded, transcribed into English, and coded using inductive-deductive approaches.

**Results:**

COVID-19 considerably impacted routine health care, mainly maternity services and immunization. Inadequate financial resources, inadequate human resources, and the lack of ventilators, ICUs, and X-ray services were the significant challenges in tackling and managing COVID-19 effectively.

**Conclusion:**

The study found that all three levels of government perform their roles and responsibilities and effectively manage the pandemic. The federal and provincial governments focused more on the plans and policy development, while the local government demonstrated greater accountability in implementing those plans and policies. Therefore, all three tiers of government need to coordinate together for preparing and communicating information in times of emergency. Besides, it is imperative to empower local governments to maintain Nepal's federal health system.

## 1 Background

In December 2019, the first case of COVID-19 caused by the novel coronavirus named as Severe Acute Respiratory syndrome Coronavirus 2 (SARS-CoV-2) was reported by officials in Wuhan, China; with the rapid spread of disease from China to other countries, it was declared as a Public Health Emergency of International Concern (PHEIC) by World Health Organization (WHO) on 30 January 2020 [1]. As of 30 August,2020 —over 1.8 million new COVID-19 cases and 38,000 new deaths were reported globally; since the start of the outbreak, a cumulative total of nearly 25 million cases and 800,000 deaths have been reported [2].

COVID-19 has been an unprecedented challenge globally. COVID-19 is a complex challenge for all governments, especially those with limited state capability [3]. Health systems worldwide are being challenged by the increasing demand for care of people with COVID-19, along with the disruption in health service delivery due to fear of COVID contraction, stigma, misinformation, and limitations on movement [4]. The alarming speed of the COVID-19 pandemic harms the global economy; the global cost of the pandemic could range from $2.0 trillion to $4.1 trillion, equivalent to a loss of 2.3% to 4.8% of global gross domestic product [5].

There was a wide variation in the national response to COVID-19 across the states of Latin America. Brazil and Mexico exhibited high heterogeneity in their subnational responses [[6], [7], [8], [9]]. There was a lack of uniform federal response and coordination between national and sub-national levels making the health system vulnerable to combat against COVID-19 pandemic. Argentina and Colombia showed consistent national guidelines with subnational variations. Bolivia, Chile, and Peru had homogeneous policies guided by centralized national policies [7]. The study emphasized that subnational responses cannot replace coordinated national policy and recommended that governments focus on evidence-based national policies while coordinating with subnational governments. The study found that the decentralization of health decision-making before the pandemic influenced the implementation of national policies. The structure of a country's government (federal or unitary) did not explain the stringency of the response. Overall, there was significant cross-national and subnational variation in public health policies across Latin America [7].

Another study examines the roles of subnational and national governments in Canada and the United States during the early phase of the COVID-19 pandemic. In the United States, state and local governments were first to take action, while in Canada, policy-making was spread out between bureaucratic institutions and provincial or federal governments, with provinces playing a significant role. The study compares the two countries across several dimensions and find that sub-national governments played a more significant role in policy development than national-level policies. However, the study highlights the uncertainty and needs for clarity regarding which level of government should be responsible for implementing policies [10].

Nepal moved from a unitary government to a federal system of government in 2015 under its constitution [11]. Nepal is a federal democratic republic governed by three levels of government: a federal, provincial, and local level [12]. In Nepal, the first case was reported on January 23, 2020, of a 32-year-old Nepali man returning from Wuhan [13]. Once the first case was identified, Nepal focused mainly on identifying and managing cases [14]. The response to COVID-19 in Nepal was substantially led and controlled by the federal government [15]. The decisions related to the pandemic response were very top-down, often patronizing, in the form of directives, guidelines, and requests to provincial and local governments. The provinces established isolation centers, testing labs, and managed medicines and other essential services. Local governments' response concentrated on establishing and managing quarantine facilities and distributing relief packages to needy people. Even though all three levels of government worked together, a systematic, centralized approach was adopted, which compromised the roles of provincial and local governments. Furthermore, the provincial and local level was not provided space to influence the response to the pandemic independently except for carrying out their routine responsibilities [15].

**Fig. 1:**
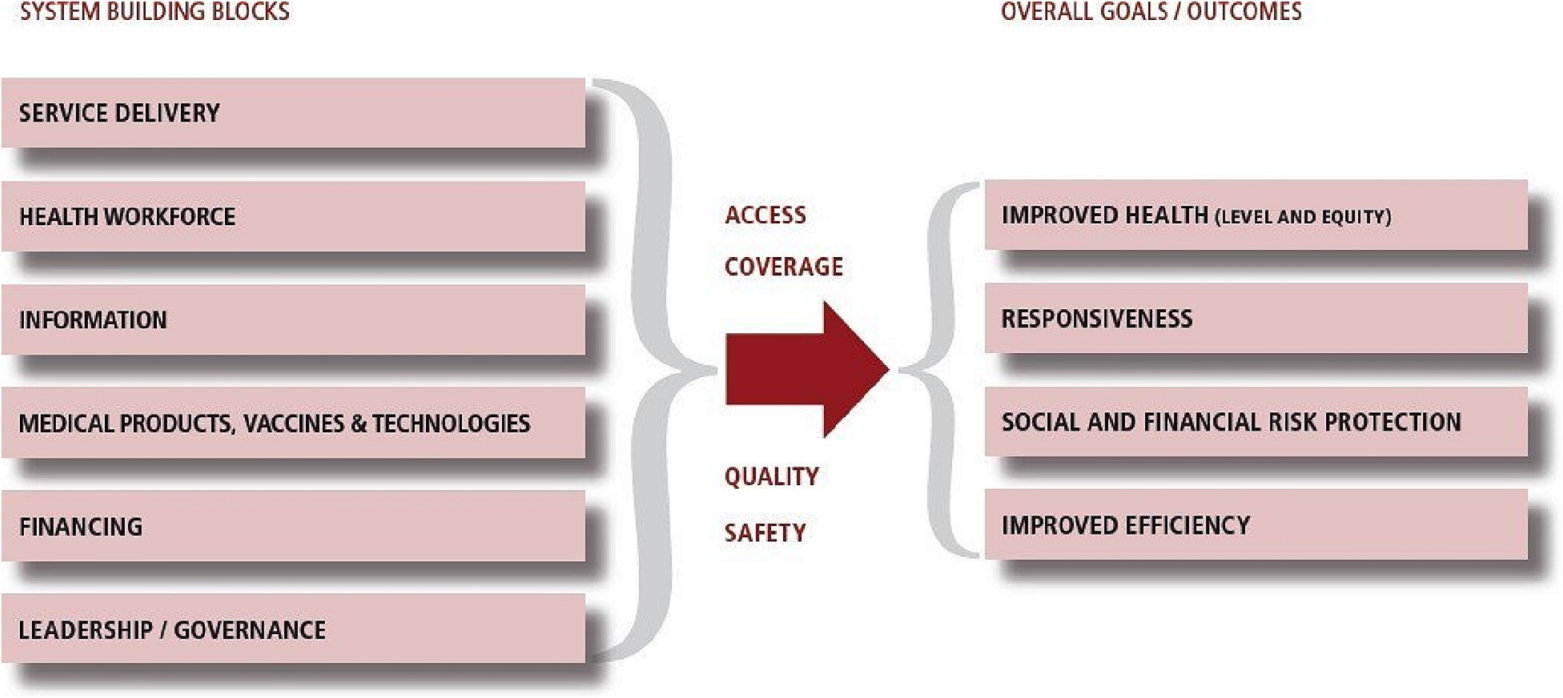
The WHO Health Systems Framework for the health care system [20].

The World Health Organization's (WHO) Health Systems Building Blocks is a classic framework used in various studies to describe the health system response to the COVID-19 pandemic [[16], [17], [18]]. As shown in Fig. 1, the building blocks are leadership and governance, service delivery, health workforce, information, financing and, logistics. The service delivery block emphasizes providing quality healthcare services, while the health workforce block focuses on ensuring a skilled and sufficient healthcare workforce. The information system block highlights the importance of reliable data for effective decision-making, and the access to essential medicines block emphasizes the equitable availability of medicines. The financing block underscores the need for sustainable funding mechanisms, and the leadership/governance block emphasizes effective management and coordination of the health system. By addressing these six building blocks, countries can strengthen their health systems and enhance their ability to respond to health challenges, including pandemics like COVID-19, and improve overall population health outcomes [19].

Two studies were conducted, one in Nigeria [16] and another in Sub-Saharan Africa (SSA) [17], using the World Health Organization's (WHO) building blocks of the health system to evaluate the preparedness and response to the COVID-19 pandemic. In Nigeria, the study revealed a sub-optimal response characterized by inadequate funding, a shortage of human resources, and insufficient infrastructure within the health system [16]. Similarly, the study conducted in SSA highlighted the long-standing challenges of abysmal health system financing and inadequate government investment, which hindered the effective functioning of the health system during the COVID-19 pandemic. These challenges not only impede the health system's ability to address the pandemic's demands but also affect the provision of other essential health services [17].

Nepal, with a poor health system due to inadequate resources, is more vulnerable. Nepal's existing public health system does not have adequate capacity to address COVID-19 or any other pandemic, for that matter [21]. Nepal, at its initial stage of the federalization process, needs more precise roles and responsibilities, coordination mechanisms, information channels, and fiscal relations for an effective response to a pandemic of the magnitude of COVID-19 [15]. Understanding and analyzing the roles and responsibilities of each health system level can play a vital role in future preparedness and response to public health emergencies like COVID-19. However, there needs to be more research analyzing the health system in the context of COVID-19. Thus, the study aims to critically analyze Nepal's health system in the context of COVID-19 response. The study contributes to identifying gaps in the health system and helps address those gaps for better preparedness, response, and management in the future.

## 2 Methodology

### 2.1 Study design

A qualitative study was conducted using phenomenological approach [22] which was involved in-depth interviews to collect the information from policymakers, health workers and stakeholders at the federal, provincial, and local levels. The objective of the study was to examine the perspectives of health workers and policy makers directly engaged in the COVID-19 pandemic response and management. To achieve this objective, a phenomenological approach was utilized, allows to describe of relevant phenomena based on the personal experiences and insights of individuals involved [23].

### 2.2 Study setting

The study was conducted in the national capital Kathmandu [24] and Karnali province of Nepal—Nepalese healthcare system is divided into federal, provincial, and local [12] governments. For federal COVID-19 preparedness and response, we collected the data under the Ministry of Health and Population, and for provincial and local level COVID-19 response, we selected Karnali province. It is one of the seven self-governed provinces of Nepal, which mainly constitute mountain land of the north and mid hills of Nepal. It is the largest province of Nepal with an area of 27,984 Km^2^, and it is the lowest-population province of Nepal with a total population of 15,70,418. Karnali province is divided into ten districts, and districts are subdivided into 79 local governments (25 municipalities and 54 rural municipalities) [25].

When the spread of coronavirus began in the country, Karnali Province was far from COVID-19 preparedness. There were only 17 ICU beds with six ventilators in the health facilities of the Jumla and Surkhet districts. The biggest hospital in the province did not have adequate ICU beds and ventilator service. The quarantine facilities in the municipalities do not meet the government's prescribed standards. There were no proper arrangements for the migrant workers returning from India—further, the geographical remoteness and shortage of human resources constraints the COVID-19 containment measures [[26], [27], [28]].

### 2.3 Study participants

The study participants in this research encompassed diverse individuals who played crucial roles in managing COVID-19 at the federal, provincial, and local levels. Representatives from institutions such as the Ministry of Health and Population, Epidemiology and Disease Control Division, National Academy of Health Sciences, and Tribhuwan University Teach Hospital were included at the Federal level. Additionally, participants from the provincial level were selected from the Ministry of Social Development, Provincial Health Directorate, Karnali Academy of Health Sciences, and front-line health workers directly involved in COVID-19 management. The involvement of local levels was ensured by the participation of the mayor/chairpersons, Chief Administrative Officer, Health Coordinators, and COVID-19-infected and recovered patients. Furthermore, stakeholders from organizations such as the Lions Club, Rotary Club, and journalists were also included in the study, reflecting the comprehensive nature of the research participants.

### 2.4 Sample size

A total of 41 interviews were conducted for this study, comprising 10 interviews from the federal level, 13 from the provincial level, and 18 from the local level. The sample size determination was based on reaching code saturation [29], which signifies the point at which no new or additional information emerged from the interviews.

### 2.5 Sampling technique

A purposive sampling [30] method was used for the selection of the participants. The participants shared common characteristics and were actively involved in COVID-19 management. By virtue of their experience, these participants were expected to provide the necessary information required by the study more effectively due to their firsthand experience in the field.

### 2.6 Data collection

The study utilized semi-structured in-depth interviews to collect the data from 41 study participants. All the interviews were conducted remotely via telephone between January to July 2021 due to the country's pandemic. An interview guideline was developed to collect the data based on the literature review and input from the experts. The tool was validated through pre-testing. Before the data collection, the research assistants were trained on the qualitative method, tools, and how to find the most appropriate and clear language to express technical terms to the team with relevant experience in qualitative methods. All the interviews were audio recorded, and verbatim notes were also taken during the interview. Each interview lasted for about 40–45 min. An iterative process [31] was used by reviewing each interview shortly after completion and adjusting subsequent interviews probing more deeply into themes emerging in earlier interviews. All the interviews were transcribed verbatim in Nepali and then translated into English. We assessed all transcripts for accuracy by cross-checking the audio recordings.

### 2.7 Data analysis

The data were analyzed using thematic analysis [32]. The initial codebook was deductively developed based on the interview guide and revised to include emerging code using an inductive approach. The Consolidated Criteria for Reporting Qualitative Studies (COREQ) guideline [33] were used in the preparation of this manuscript.

## 3 Ethical consideration

The study was approved by the ethical review committee at Kathmandu University School of Medical Sciences (Ref: 127/20; KUSMS IRC). The participants were informed about the objectives and methodology of the study. Participants were asked to participate voluntarily. Participants were informed that they had a total right not to participate or discontinue the study at any time during the study.

## 4 Results

Among the 41 respondents interviewed, 10 were from the Federal level (4 health workers, 3 policymakers, 3 stakeholders), 13 from the Provincial level (5 health workers, 5 policymakers, 3 stakeholders), and 18 from the local level (6 health workers, 10 policymakers, 2 stakeholders). Most (85.4%) were male, and only 6 (14.6%) were female. The mean age of the respondents was 40.1 years (SD: 10.5). Table 1 shows the demographics of the respondents who participated in the study.

**Table 1:** Socio-demographic characteristics of the participants.

Characteristics	Categories	Frequency (%)
Age (years)Mean ± SD	Continuous	40.1 (10.5)
Gender	Male	35 (85.4%)
	Female	6 (14.6%)
Level of government	Federal	10 (24.4%)
	Provincial	13 (31.7%)
	Local	18 (43.9%)
Occupation	Health worker/Health manager	15 (36.6%)
	Policy maker	18 (43.9%)
	Stakeholder	8 (19.5%)

Using thematic analysis, the findings from the study were categorized into six themes according to the WHO's building blocks of the health system.

### 4.1 Leadership and governance

The three tiers of the government had their own roles and responsibilities in response to COVID-19 pandemic with more responsibility to the local governments. The federal and provincial level were more concentrated on formulation of the various plans, policies, guidelines, and directives, and facilitated in their implementation. And the local government was more responsible in the implementation and followed the plans, policies and guidelines provided by the federal and provincial government.

*“We developed guidelines for most of the things… We found the active leadership of the provincial government. Likewise, the local government was also active. There was engagement of both provincial and local governments in things like quarantine management, contact tracing, establishment of hospitals. They both led the situation in an effective manner. There was good intention from both the government in managing COVID. The stakeholders were also actively involved under the leadership of the provincial and local government.”*

*(Federal level policy maker; p1; Kathmandu)*

*“The Federal government is leading COVID-19 management. It has been developing policy and guidelines. Central government is like our parenting body that is why we are following the policies developed by it as much as we can regarding the things as how to make it understandable to people, regarding lockdown…..In the context of COVID management, local government has major roles in implementation rather than central and provincial government. That's why local government is active for implementation.”*

*(Local level policy maker; p6; Salyan)*

However, lack of clarity on roles and responsibilities of each level of government has created problems in implementation and management.

*“The local government says that it has to be done by the provincial government while the federal government says that since the system has undergone a local level it is to be done by them. So we are in a dilemma regarding our job description.”*

*(Provincial level policymaker; p3; Kalikot)*

There is coordination between the local, provincial and federal level of government through different means of communication and information. Regarding the COVID-19 management part federal, provincial and local all of them are fulfilling their responsibility accordingly and with the combination of these bodies the things are proceeding well.

*“Now from federal level to province level to local level communication and coordination is being done. Province government communicates every activities of federal government and it communicates that information to the local level trough hard copy or email or soft copy or telephone or by the use of different media they are coordinating with local levels….”*

*(Provincial level policy maker; p2; Dolpa)*

However, lacking coordination between federal and provincial governments was expressed by the stakeholder of the Provincial level.

*“We see the Nepal government at the central level, provincial level and local level, and what we have found is that there is no proper coordination between the federal government and provincial government.”*

*(Provincial level Stakeholder, p2; Surkhet)*

### 4.2 Service delivery

Most of the health workers in this study agreed that there was a significant impact of COVID- 19 on routine health care with mostly maternity services, immunization as the health facilities were mainly providing COVID related services. Additionally, the health facilities were unable to provide its usual routine health services due to the lockdown. And there was fear of contracting COVID from the health personnel among the people. As a result, pregnant women did not visit the health facilities for ANC checkups and the people with chronic diseases did not get timely checkup, follow-up and medication. Consequently, there was an increase in the number of home deliveries and neonatal deaths. Likely, there was also an increase in immunization drop-out rate among the children. Along with this, due to more number of COVID-19 patients, even non-COVID critically ill patients were not getting ICU and ventilators.

*“….. Due to COVID-19, we have postponed the surgery on our regular plan… Due to the pressure of COVID-19 patients, we could not keep non-COVID critically ill patients in ICU and ventilators…”.*

*(Federal level Front line HW; p6; Bhaktapur)*

*“For the people with chronic diseases, they are not getting timely medication, timely checkup, and timely follow up and it has affected them. In case of pregnant mother, they don't get timely ANC checkup which has added up for the increased neonatal mortality.”*

*(Provincial Level Front Line HW p9; Jumla)*

*“There has been a big influence on regular health service because of COVID. People stay at home because of fear of getting infected of COVID-19 while visiting health centers. That's why it has created a negative impact. The number of people who are having home delivery has increased because of lockdown which even lead to death of 1/2 newborns along with it people also don't visit for ANC and dropout in routine immunization has also increased.”*

*(Local level, policy makers, p8, Dailekh)*

At all three levels, institutional isolation was preferred over home isolation because at institutional isolation, there was an arrangement of food and accommodation. The patients would have better and timely care and treatment from the health workers. Moreover, in the context of inadequate space, home isolation was difficult. So, institutional isolation was better than home isolation.

*“In Jumla, the traditional houses have no separate toilet, kitchen, bathroom and almost the entire family lives in 1/2 rooms. Moreover, there is no proper ventilation so home isolation is difficult in the context of Jumla. Initially we encouraged for home isolation but it was not effective and due to this, we decided to keep people in institutional isolation.”*

*(Provincial Level, Hospital Manager; p7, Jumla)*

However, the governments have managed the cases of home isolation. Those cases of home isolation were constantly monitored by the health workers. They followed up the cases for their health conditions either through the phone calls or visit to the patient's house on a regular basis. At local level, the health facility also provided the essential medicines and corona prevention, control and treatment kit to home isolation patients. The federal level hospital was providing counselling to the people living in home isolation.

*“For home isolation, if a person tests positive then it will be recorded in rural municipality and later on as chief of health center and other health service provider relating to COVID will call them time to time to get informed about their health condition.”*

*(Local level policy maker; p6; Salyan)*

At local and provincial level, the contact tracing was not done effectively mainly due to inadequate human resources and lack of orientation and training to the existing human resources. The federal level was also back in the contact tracing process due to various circumstances.

*“...The contact tracing is not conducted as per the guidelines. As we look at Salyan, it has not been done effectively because we don't have enough human resources. There is scarcity of human resources and these should be provided. They should be provided with proper training on contact tracing after which the situation could be better.”*

*(Provincial Level Policy Maker, p4, Salyan)*

*“Under the leadership of a medical officer, we have formulated and mobilized a CICT team and trained the team about it... But the doctor has returned to his/her own sanctioned place and we are not able to provide CICT orientation to the new doctor. So, presently we could not do contact tracing effectively like before.”*

*(Local Level Policy Maker, p8, Dailekh)*

### 4.3 Human resources

The Karnali Province and its local levels had insufficient health personnel. In addition, specialist human resources were inadequate for critical case management. At the local level, though the federal government has provided a certain amount for only three months, there were not enough applicants. This could be due to no secure job. As a result, the local level was unable to afford specialized health personnel. Likely, at provincial level, there was only one doctor available for providing the services to 25/28 patients.

*“There is scarcity of doctors and there are hardly 5 doctors out of district and there are no doctors on local level and in each hospital, 25/ 28 patients are admitted daily and for these patients there is one medical officer who can hardly give medicines in sufficient manner.”*

*(Provincial Level Policy Maker, p3, Kalikot)*

Additionally, at the provincial level, there was a shortage of skilled human resources. Despite having ventilator and oxygen plants in the hospitals, they were not in function because of lack of human resources with the required skills to operate those equipment.

*“The provision for the availability of oxygen plants in every hospital of the district is not complete but still is in process. We have beds, we have ventilators but the human resources who have skills and abilities to operate those available ventilators are still inadequate. For this, proper training should be given and these all are in process”.*

*(Provincial level Policy Makers, p2, Surkhet)*

The inadequate number of human resources was due to difficulty in human resource mobilization after the staff adjustment. After the staff adjustment, staff were adjusted at the federal, provincial and local level. The sanctioned posts at provincial and local were vacant and there was no vacancy announcement from the concerned authority. So, the local level, with its own resources, temporarily recruited the health workers for effective service delivery. And for the federal government, it was difficult to mobilize staff in this situation of a pandemic.

*“The sanctioned posts are not well fulfilled and because of staff adjustment, health workers who were working here also moved. So as the staff are not coming after additional staff adjustment we are providing service by hiring temporary staff. We do have a great challenge of lack of human resource but still we are managing by hiring temporary staffs….I have been reporting time and again about the vacant post of health workers to concerned authority but still it's not being heard from any places...”*

*(Local level Policy Makers, p8, Dailekh)*

For the motivations of the frontline health workers, the Provincial and the local government were providing incentives and allowances such as communication allowance, hazard allowance. However, health workers expressed their opinion on security rather than the 50% hazard allowances.

*“Motivation program has been initiated in Karnali provinces. Doctors in Surkhet are provided with 50 % allowance and 135% as in Humla/Dolpa as per their level and for other health service providers and health officers, the money grant of 4000 is made available, which has provided support for motivation…”.*

*(Provincial level policy Makers, p1, Salyan)*

*“They are receiving hazard allowance as per the decision of our local executive. They are receiving a daily allowance of NPR 650 per day. We are also providing them with PPE sets, masks and sanitizer. Rural municipality will be held accountable if they encounter with any problem and get sick.”*

*(Local level Policy Makers, p9, Dolpa)*

### 4.4 Information system

There was a regular information flow mechanism between the three tiers of government. The local government reported community level COVID data to the provincial level daily via online which is then reported to the federal level. The federal and provincial governments collected different COVID-19 related information on a daily basis. However, the quality of data was a major concern.

*“Every district of our local level reports on the number of patients admitted in isolation centers, numbers of patients under treatment, number of patients who have recovered and of the ones who lost their lives during treatment to DCCMC and health service offices. We compile all this information from the district, and report it to the Ministry of Social Development, and in CCMC and Ministry of Health and Department of Health Service...”*

*(Provincial Level; Policy Maker, p5, Dailekh)*

*“We are reporting our works and its findings daily via online. We collect information regarding health situation, COVID-19 condition from the 7 health service centers of 6 different wards of Thatigadh RM and this report is handed over to health office, Dailekh. DAO is informed about it through HO. Similarly central will get informed through DAO. We exchange this information on time in this way.”*

*(Local Level Policy Makers, p8, Dailekh)*

*“There are large number of patient in local level and we are having easy access about the data of patient from local level but the reliability of the data is a major concern.”*

*(Federal Level Front Line Health Workers, p3, Kathmandu)*

In Karnali province, due to technical issues with the internet and lack of electricity which caused problems with data entry and reporting. However, they reported either by phone call or by using a mobile data pack. And lack of technical competency among the staff has resulted in low quality of data.

*“...Lack of electricity facilities has created difficulty in reporting system and sometimes even due the lack of technical knowledge on reporting process among the staffs has also created the problem in reporting.”*

*(Provincial Level Policy Makers, p5, Dailekh)*

*“Health service providers from the lab are reporting via online but we are facing some problems regarding reporting. Due to internet problems we are not able to report at time. Sometimes we have to send data of 100 people collectively but due to internet problems we are using mobile data. Still the online data entry from computers have stopped completely. Sometimes there is a network problem and even at times of emergency we cannot make a call. It is getting somehow difficult because of it.”*

*(Local Level Policy Maker, p10, Jumla)*

### 4.5 Financing

At the local level, the government has initiated budget allocation for the COVID-19 management. However, the budget was inadequate. As a result, corona crisis funds were established at the municipal level in order to handle and respond to COVID-19 effectively. The budget provided from the federal and provincial government and other financial aids were deposited in the fund and the local government was using those funds for the COVID management and other immediate financial problems.

*“For controlling the COVID-19 we have separated one crore as per our capacity so we have been managing from it. But still the budget we are receiving from the central government is still not enough. It is causing us problem for COVID-19 management.”*

*(Local Level Policy Makers, p2, Dolpa)*

*“The COVID-19 fund has been established at the local level where the budget sent from the central and provincial government along with the fund given by any other parties is deposited but we have quite a low amount in it. Last year, province had sent us 15/20 lakhs amount but we don't have any idea about the amount of budget provincial and central government is sending us this year.”*

*(Local Level Policy Maker, p6, Salyan)*

The COVID-19 response fund was primarily managed by local governments. In the context of managing the expenses for the COVID-19 response, the deficit budget was maintained through the fund transfer mechanism. The approved budget for the developmental sector as well as other programs budget which could not be implemented in this year due to COVID-19 was transferred to the corona crisis fund.

*“We are receiving a budget from both province and center and along with it the executive body of the rural municipality had decided to deposit and operate those budgets of other different headings which are not being operated due to lockdown also into the disaster management fund for control of COVID-19. …We were given a budget from the province both last year and now. We are using the budget of rural municipality under different headings by diverting those amount into it.”*

*(Local Level Policy Maker, p9, Dolpa)*

However, the provincial government faced some challenges like late release of budget and no proper guidelines on budget expenditure. Due to these, it was difficult in timely procurement of the necessary materials for COVID management.

*“…….We have the necessary budget but we did not find necessary materials in the market. No medicines were found so it was very difficult to manage COVID-19 this time…”.*

*(Provincial Level Policy Makers, p5, Dailekh)*

### 4.6 Logistics

The Provincial and local government were able to manage the necessary resources. PPEs and other essential equipment were scarce in the early days of COVID-19. Later, the local government was able to obtain the PPEs, oxygen cylinders, and other required equipment using its own resources. And the provincial government was also prepared from the first wave and was having enough stocks. So this was no longer a major concern. However, the federal government was having problems in the supply of the PPE and testing kits due to a shortage of the materials and the need to import from outside.

*“We have a shortage of PPE and a test kit for PCR as we need to buy it from outside. As it does have high demand, we are having the condition of untimely supply of these things.”*

*(Federal level Policy Makers, P3, Kathmandu)*

*“As we were preparing from the first wave and as we were given donations, we did not face much crisis of other equipment as compared to oxygen. We assumed there might be a second wave coming and thus we prepared from the first wave. That's why, we don't face difficulties in PCR, masks and kit till now and we have enough stock as well.”*

*(Provincial Level Health Care Manager, p7, Jumla)*

*“I have not experienced any sort of deficiency in our municipality because municipality have been easily providing us the materials that we have demanded relating with COVID- 19.”*

*(Local Level Policy Makers, P7, Surkhet)*

There was remarkable support from different I /NGO, private sectors, developmental partners working at local, provincial and federal level. They supported the government with necessary logistics, equipment and testing kits.

*“During the second wave, there was a shortage of testing kits. So we [Lions Club 325] bought kits of worth $40,000 during this pandemic, and distributed it to the diagnosis centers where needed.”*

*(Federal Level Stakeholder, p5)*

*“Different organizations working in Karnali Pradesh and different private organizations also helped us by providing oxygen plants, oxygen concentrators, oxygen cylinder management and different health related equipment…”.*

*(Provincial Level Policy Makers, p1, Surkhet)*

*“USAID AND UNICEF have been helping us. UNICEF had helped us by providing oxygen concentrator, pulse oxymeter whereas USAID had provided us with medicines, masks, sanitizer and PPE set.”*

*(Local Level Front Line HW, p11, Dailekh)*

The Karnali Provincial and local government was having difficulty in handling this pandemic situation with the existing physical infrastructures. The existing infrastructure in a remote part of Karnali was inadequate. Due to the lack of ventilators, ICU and X-ray services, health facilities were unable to manage the critical cases in an effective manner.

*“Looking at the numbers of diseased patients, we have a sufficient isolation center. We had started off with 15 beds which was later increased to 40 and we faced difficulties then to some extent and in the midway, we placed the severely symptomatic patients which made us easier. We don't have ICU, HDU and they are not in the district as well. It is difficult to bring in Kalikot and yes, ICU, HDU are not enough. This was not made possible by Nepal government or by us as well.”*

*(Provincial Level, Front Line HW, p10, Kalikot)*

*“We do have 3 health centers in total but we don't have oxygen management in any of them for now. Similarly we also have x-ray and video x-ray machines. Likewise we also don't have bed and test kits. Hence, these things are to be fulfilled.”*

*(Local Level Policy Makers, p5, Dolpa)*

### 4.7 Lesson learnt

Majority of the policy makers learnt that early preparedness is required for better COVID management. The three tiers of government should be prepared in terms of human resources, the infrastructure and other necessary resources.

*“The necessity of redefining our approaches and infrastructures, modification of acts is what we learnt from this pandemic along with this we have also learnt about the need of framework for legal provisions and necessity of readiness in order to tackle with similar kind of pandemics…”.*

*(Federal Level Policy Makers, p2, Kathmandu)*

*“Mainly we weren't properly prepared, our preparedness was not enough. Though we talked about the ratio of health institutions, nurse patient ratio, doctor patient ratio, the human resources and equipment in the first wave, the oxygen preparedness was not done suddenly in the second wave. We were not prepared properly. So, we should develop the health sector by considering equipment, manpower and we must increase the capacity to tackle the disaster. If we have 100 patients in PHCC level, we can handle about 50 patients during a disaster. We should plan thoroughly and we must strengthen our capacity.”*

*(Provincial Level, Health Care Manager, p7, and Jumla)*

*“It's learnt there should be early preparedness from all tiers of government by targeting the health of individuals as there may be these kinds of problems in the coming future too. If it is not done then it would lead to a fearful situation as loss of people's lives. That's why the major thing is there should be the management of hospitals with beds and specialized doctors even in the lower level…”.*

*(Local Level Policy Makers, p6, Salyan)*

### 4.8 Recommendation

The policymakers and health workers recommended that each level of government should attend the competencies to work independently in their concerned area with major focus on local level. There is a huge responsibility of the local level government in the implementation process during this pandemic. A proper coordination and clarity on the roles and responsibilities in each level of government is needed for the effective COVID-19 management.

*“It has come to the point of realization regarding the competency and need of each level of government, how the local level run without the province can and how can federal work without the province. So on the basis of this realization, responsibility, adjustment, human resource and capacity building should be addressed accordingly…”.*

*(Federal Level, Policy Makers, p2, Kathmandu)*

*“The government should not work through a blanket approach. While formulating a plan, things such as: background of the province, geographical difficulty, provincial capacity and their income and others should be taken into consideration. The matching point or logistic point or the matching point in catchment area, if these all are possible, then all Nepalese or all citizens will have access to health service as mentioned in constitution.”*

*Provincial Level Policy Makers, p2, Surkhet*

*“Local government would be competent if it is provided with technical support and power/right to manage the health emergency so that it could move forward by deciding their risk on its own.”*

*(Local Level Policy Makers, p5, Dolpa)*

*“The local government should be very active and technically competent as it has a more significant role in the implementation process. Depending upon the federal level might not always work. So, the local level government should stay active by managing human resources. Along with it there should be adequate development of physical structures. Likewise, there should be coordination between the federal, provincial and local government.”*

*(Local Level, Front Line HW, p13, West Rukum)*

## 5 Discussion

### 5.1 Leadership and governance

The three tiers of the government had their own roles and responsibilities in response to COVID-19 pandemic. The federal and provincial level were more concentrated on formulation of the various plans, policies, guidelines, and directives, and facilitated in their implementation. And, the local government was more responsible in the implementation and followed the plans, policies and guidelines provided by the federal and provincial government. However, there was poor coordination and unclear roles and responsibilities which led to miscommunication. So, things got overlapped regarding work between the local, provincial and federal level. This finding is also echoed by the previous several studies that found that the poor coordination between the three tiers of the health system (federal, provincial and local) was an important gap in the health service preparedness in COVID-19 management [34]. Since the functions and responsibilities of the three levels of government were not clearly defined and there was no effective mechanism for coordination and collaboration, there were barriers in COVID-19 management [35]. The federal and provincial governments passed protocols, guidelines, but their immediate implementation was not satisfactory. The local government complained regarding the circulation of those guidelines, protocols. As per the local government, the federal and provincial governments have not done much to circulate those protocols, and guidelines [36].

### 5.2 Service delivery

The COVID pandemic had its impact on routine health care. People avoided going to the health facility because they were afraid of contracting COVID from the health personnel. As a result, home delivery and immunization drop-out rates had increased. This result is supported by the previous study [37]^,^ where the lockdown has caused disruption in the routine health care services such as maternal health, immunization and chronic diseases. There was an increase in home deliveries and few neonatal deaths.

The study conducted in a joint initiative of the Integrated Health Information Management Section, Management Division, Department of Health Services, Ministry of Health and Population and Nepal Health Sector Support Program (NHSSP) suggested that there was an initial impacts of COVID-19 in all service utilization [38]. During the initial lockdown, there was a medicine shortage, delivery services were unavailable, and ambulance services too were unavailable for many days. Likely, there was more number of maternal deaths in COVID-19 months. The participants in our study reported that at all the three levels, both institutional, and home isolation services were properly managed. There was an arrangement of food and accommodation as well as timely care and treatment from the health workers. However, a previous study had contrast finding where the participants reported that the isolation and quarantine services were inadequate to provide good care for COVID-19 patients. Particularly, political connections and those in positions of power and affluence used the medical facilities, leaving the poor and underprivileged without care [37].

In our study, at all level, the contract tracing was not done effectively mainly due to inadequate human resources and lack of orientation and training to the existing human resources. Similarly, a rapid assessment found that it was easy to conduct contact tracing in rural areas since it was simple to diagnose the cases but it was more difficult in urban areas [35].

### 5.3 Human resources

In our study, the participants reported that the existing human resources were inadequate which appeared as a major challenge in providing COVID-19 services. These findings have been supported by the previous studies [34,39,40] where the existing health workers were divided to provide both COVID-19 and non-COVID health care services. The doctors were insufficient in number and there was no additional health workers for substitution. The government was unable to increase the number of the health workers due to the short term vacancy announcement which went unfilled [34].

Moreover, there was a shortage of specialized human resources for critical case management in the provincial and local level. After the staff adjustment, staffs were adjusted at the federal, provincial and local level. It was difficult to mobilize staff from one level to another level of government in this situation of a pandemic which posed a significant impact on COVID-19 management. Additionally, there were more vacant posts at provincial and local level. There was no vacancy announcement by the concerned body for the fulfillment of sanctioned posts. This has also caused problems in human resource management.

Most participants in our study expressed that the provincial and the local government were providing incentives and allowances such as communication allowance, hazard allowance as the motivations for the frontline health workers, However, a study conducted in 3 public hospitals of Eastern Nepal had a contrast finding. The participants stated that there was an unfair lack of appreciation and compensation for their efforts, which left them less motivated to work during this difficult period [40].

### 5.4 Information system

There was a regular online information flow mechanism between the three tiers of government. The local level reported on the data of new cases, treated cases, recovered cases, and deaths to the provincial level, which was then reported to the federal level. The federal and provincial government also updated the local level on the current situation. There was two-way communication. However, there were some challenges in data entry and reporting from local and provincial level to the federal level such as technical issues with the internet, electricity and inadequate technically competent human resource. The study conducted in a joint initiative of the Integrated Health Information Management Section, Management Division also had similar finding. In the COVID-19 month, there was no discernible difference in the HMIS reporting's timeliness. However, the main factors affecting the performance of Maternal and Perinatal Death Surveillance and Response during the pandemic period were determined to be inadequate institutionalization of systems, limited access to internet resources, and a lack of human resources and monitoring mechanisms [38].

### 5.5 Health financing

The COVID-19 response fund was primarily managed by local governments. Budgetary support was also granted by the federal and provincial governments to local governments for COVID19 management. But the budget grant was insufficient. As a result, corona crisis funds were established at the municipal level in order to handle and respond to COVID-19 effectively. The limited financial transfer has been the major challenge to local levels in combating Covid-19.[36] There were some challenges in budget expenditure such as late budget release and no clear instruction on the budget expenditure process due to which the procurement and the implementation process could not be accomplished on time.

### 5.6 Logistics

PPEs and other essential equipment were scarce in the early days of COVID-19. Later, the local and the provincial government was able to obtain the PPEs, oxygen cylinders, and other required equipment using its own resources, so this was no longer a major concern. There was remarkable logistics support from I/NGOs, UN agencies, EDPs working at the local, provincial and federal level. This finding is also in accordance with a study done which focused on the rapid assessment of COVID19 related policies, guidelines, and directives issued by MoHP and its agencies and its implementation practices [35]. During the early stages of COVID-19, it was challenging to manage the cases in Karnali Province because of limited PPEs and other necessary equipment. Later, this was no longer a serious issue due to the support of numerous government and non-governmental organizations.

However, the existing infrastructure in a remote part of Karnali was inadequate. Due to the lack of ventilators, ICU and X-ray services, health facilities were unable to manage the critical cases in an effective manner. These findings are supported by the studies [34,40] where hospitals had to run the services in the limited resources such as isolation beds, ICUs and ventilators, oxygen plant, PPEs etc. The existing buildings, other infrastructure and equipment were reportedly made worse by the COVID-19. Insufficient capacity for lab testing and contact tracking were the major issues as expressed by the health workers [34].

### 5.7 Strengths and limitations

The research involved various participants, such as policymakers, healthcare workers, stakeholders, and individuals who have recovered from COVID-19. These participants represent different levels of government, resulting in diverse opinions. The study was conducted in Karnali province and its local levels in Nepal. The region is characterized by various factors, including limited health and transportation infrastructure, a shortage of medical staff, insufficient testing capabilities, and the return of family members from India. Additionally, the area has mountainous and hilly terrain [25,41]. As a result, these study's findings may not be applicable to other regions of Nepal. However, our research findings can be utilized to shape forthcoming policies and programs, specifically focusing on enhancing preparedness and managing disaster and health emergencies in Nepal.

## 6 Conclusion

The study concludes that all three tiers of government perform their roles and responsibilities in responding and managing COVID-19 pandemic. However, the local level was more significant role in the implementation process. Therefore, it is essential to strengthen the local level government to ensure the sustainability of Nepal's federal health system. Additionally, effective coordination among all three tiers of government is essential for preparedness and communication of the information during the time of emergency.

## Funding

The study was partially funded by Nepal Health Research Council to implement the study under PG Health Research Grant 2021.

## Availability of data and materials

The interview transcripts generated and analyzed during this study are not publicly available to maintain the confidentiality of the participants but are available from the corresponding author on reasonable request.

## Authors' contributions

Conceived and designed the study: BSK, BMK and AS.

Tool translation to Nepali: BSK, AT, and BA.

Facilitated data collection in the field: BSK, AT, and RD.

Data transcription and coding: BSK, NRD, RD and KA.

Data analysis: BSK, NB, BA, AT, and KA.

Writing an original draft and editing of the manuscript: BSK, and BA.

Critical revision of the manuscript: BMK, AS, DX, BBS, BB, and RKM.

All authors have read and approved the manuscript.

## Declaration of Competing Interest

The authors declare that they have no competing interest.
