# Supplementary material for: A critical analysis of health system in Nepal: Perspectives based on COVID-19 response
Source: Dialogues Health. 2023 Jun 10;3:100142. doi: 10.1016/j.dialog.2023.100142 (PMC10257514; doi:10.1016/j.dialog.2023.100142)
Supplement: Supplementary material [file mmc1.pdf]

In-depth Interview (Guide)

1. Interview Guide (Federal Level)

**A Critical Analysis of Health System in Nepal; Perspectives based on COVID-19 response**
Preamble:<br>Hello my name is Bihari Sharan Kuikel, a master's student in the Department of Public Health at Kathmandu University, Dhulikhel. This study seeks to critical analysis of Health System of Nepal Perspectives based on COVID-19

**Topic**	**Questions**
Background	Could you tell me about yourself?<br>Name, Age, Designation, Experience (years)
1. Leadership & Governance	1. Were there existing public health emergency contingency, preparedness, and response plans?<br><br>2. Who is leading/managing the preparedness and response activities?<br><br>• Has a national emergency response committee been activated?<br>• What are its leadership, accountability, and role & responsibilities, and how is it functioning?<br><br>3. Is emergency legislation in place? Are appropriate delegations of authority in place?<br><br>4. How is coordination between the ministry of health, other sectoral ministries, provincial and local government, working?<br>• Has the government engaged technical experts and research networks?<br>• How well is the government doing in regard to providing guidance and planning assumptions to partners?<br><br>5. What is the role being played by different types of international agencies?<br>• Is the government able to lead the coordination of partners involved in response operations?

	• How is this being done?<br><br>6. What is the level of centralisation of the preparedness and response activities? Is anything being done to increase or decrease the decision space of local managers? What are the mechanisms for coordinating and augmenting district and national responses?
2. Service delivery	1. What has been done to strengthen the ability to identify, isolate, and care for infected patients?<br>• What are the strategies in place to limit community transmission?<br>• Is there any existing policy on isolation for confirmed cases either at institution or at home?<br>2. What has been done to strengthen the quarantine services?<br>• How were the international arrivals and migrant workers managed?<br>3. What activities have been done for testing and contact tracing?<br>• Are there any standardized protocols for testing of suspected and confirmed COVID-19 case?<br>• What is the procedure for contact tracing of confirmed case?<br>4. Have risk assessments been undertaken in respect of infection prevention and control activities at all levels of the healthcare system?<br>• Have referral pathways been defined?<br>• Have there been any changes in the provision of other services?<br>• Has there been any change in how the private sector is used?
3. Information and communication system	1. To what extent is health system relying on existing surveillance and management information systems at national level?<br>• Have new information systems been constituted?<br>• How are they being used?<br>2. Has there been a special focus on communicating with travellers, for example through airlines, travel agents, and tour operators?<br>3. What is the communication channel between the different levels of government?<br>• Are there any guidelines on reporting COVID-19 cases?

	• How is the information being disseminated? (traditional, social media, hotlines?)<br>4. Have risk communication been undertaken in respect of timely data analysis and operational decision making?
5. Health Workforce	1. Do you think there are adequate frontline health workers to handle the COVID-19 crisis? If not, why?<br>2. What is being done to create surge capacity when needed?<br>3. What trainings and preparedness activities are being undertaken with the workforce?<br>• Are all avenues to take advantage of remote training of staff by radio, digital media, etc. exploited?<br>• Are plans being put in place to protect frontline workers?<br>• Has the federal government provided technical support to the provincial and local government? If yes, what kind of support and human resources involved?
6. Finance	1. Is there an estimate of additional funding needs for COVID-19 emergency preparedness and response measures?<br>• Is there any government contingency fund, government budget reallocations, donor support, etc?<br>• How much of the needed additional funding has been mobilised and how much more is expected?
7. Supplies, logistics and infrastructures	1. How were the logistics and essential supplies managed?<br>• Do you think the current supplies and logistics are sufficient?<br>• Are timely activities being undertaken to build up stocks of drugs, consumables, and personal protective equipment (PPEs)?<br>• Are emergency procurement and distribution plans being developed (or in place)?<br>2. What has been done to strengthen the infrastructures of the health institutions for providing quality health services?<br>• Are adjustments being made to infrastructure (e.g. treatment centres or units, upgraded or new facilities, preparation of specialist centres)?

Recommendations & Way Forward	1. What are the limitations and challenges that you have experienced in managing COVID-19 cases?<br><br>2. What should be the way forward in terms of preparedness and response to COVID-19 or similar epidemic in future?<br><br>3. Would you like to express on any other issues that are left in our discussion?

2. Interview Guide (Province Level)

A Critical Analysis of Health System in Nepal; Perspectives based on COVID-19 response

Preamble:
Hello my name is Bihari Sharan Kuikel, a master's student in the Department of Public Health at Kathmandu University, Dhulikhel. This study seeks to critical analysis of Health System of Nepal Perspectives based on COVID-19

Topic	Questions
Background	Could you tell me about yourself?<br>Name, Age, Designation, Experience (years)
1. Leadership & Governance	1. Were there existing public health emergency contingency, preparedness, and response plans?<br><br>2. Who is leading/managing the preparedness and response activities?<br><br>• Has an emergency response committee been activated?<br>• What are its leadership, accountability, and role & responsibilities, and how is it functioning?<br><br>3. Is emergency legislation in place?<br><br>4. How is coordination with federal and local government working?<br>• What is the level of centralisation of the preparedness and response activities?

	5. What is the role being played by different types of stakeholders?
2. Service delivery	1. What has been done to strengthen the ability to identify, isolate, and care for infected patients?<br>• What are the strategies in place to limit community transmission?<br>• Is there any existing policy on isolation for confirmed cases either at institution or at home?<br>2. What has been done to strengthen the quarantine services?<br>• How were the international arrivals and migrant workers managed?<br>• What are the strategies in place for home quarantine?<br>3. What activities have been done for testing and contact tracing?<br>• Are there any standardized protocols for testing of suspected and confirmed COVID-19 case?<br>• What is the procedure for contact tracing of confirmed case?<br>4. Have risk assessments been undertaken in respect of infection prevention and control activities at all levels of the healthcare system?<br>• Have referral pathways been defined?<br>• Have there been any changes in the provision of other services?
3. Information and communication system	1. What is the existing surveillance and management information systems at province level?<br>• Have new information systems been constituted?<br>• How are they being used?<br>2. Has there been a special focus on communicating with travellers, for example through airlines, travel agents, and tour operators?<br>3. What is the communication channel between the different levels of government?<br>• Are there any guidelines on reporting COVID-19 cases?<br>• How is the information being disseminated? (traditional, social media, hotlines?)<br>4. Have risk communication been undertaken in respect of timely data analysis and operational decision making?
4. Health Workforce	1. Do you think there are adequate frontline health workers to handle the COVID-19 crisis? If not, why?

	2. What trainings and preparedness activities are being undertaken with the workforce?<br>• Are all avenues to take advantage of remote training of staff by radio, digital media, etc. exploited?<br>3. Can you tell us what motivates the health workers to work amidst the fear of COVID-19?<br>• Are plans being put in place to protect frontline workers?<br>• Do they comply with the need to avoid risky gatherings and transport?<br>• Is there any provision for motivation of health workers such as incentives, insurance etc?<br>• Can you tell us something about discrimination or stigma faced by frontline health workers during this COVID 19?<br>• In such situation, is there any provision of psychosocial support to them?<br>4. What is the role of health workers in preparedness and response activities?
5. Finance	1. Is there an estimate of additional funding needs for COVID-19 emergency preparedness and response measures?<br>• Is there any provincial contingency fund budget, donor support, etc?<br>• How much of the needed additional funding has been mobilised and how much more is expected?
6. Supplies, logistics and infrastructures	1. How were the logistics and essential supplies managed?<br>• Do you think the current supplies and logistics are sufficient?<br>• Are timely activities being undertaken to build up stocks of drugs, consumables, and personal protective equipment (PPEs)?<br>• Are emergency procurement and distribution plans being developed (or in place)?<br>2. What has been done to strengthen the infrastructures of the health institutions for providing quality health services?<br>• Are adjustments being made to infrastructure (e.g. treatment centres, quarantine and isolation centers, laboratory services, preparation of specialist centres)?
Recommendations & Way Forward	1. What are the limitations and challenges that you have experienced in managing COVID-19 cases?<br>2. What should be the way forward in terms of preparedness and response to COVID-19 or similar epidemic in future?

	3. Would you like to express on any other issues that are left in our discussion?

3. Interview Guide (Local Level)

**A Critical Analysis of Health System in Nepal; Perspectives based on COVID-19 response**
Preamble:<br>Hello my name is Bihari Sharan Kuikel, a master's student in the Department of Public Health at Kathmandu University, Dhulikhel. This study seeks to critical analysis of Health System of Nepal Perspectives based on COVID-19

**Topic**	**Questions**
Background	Could you tell me about yourself?<br>Name, Age, Designation, Experience (years)
1. Leadership & Governance	1. Were there existing public health emergency contingency, preparedness, and response plans?<br><br>2. Who is leading/managing the preparedness and response activities?<br>• Has an emergency response committee been activated?<br>• What are its leadership, accountability, and role & responsibilities, and how is it functioning?<br>3. How is coordination federal and provincial government, working?<br>4. What is the level of centralisation of the preparedness and response activities?<br>5. What is the role being played by different types of stakeholders?
2. Service delivery	1. What has been done to strengthen the ability to identify, isolate, and care for infected patients?<br>• What are the strategies in place to limit community transmission?

	• Is there any existing policy on isolation for confirmed cases either at institution or at home?<br>2. What has been done to strengthen the quarantine services?<br>• How were the international arrivals and migrant workers managed?<br>• What are the strategies in place for home quarantine?<br>3. What activities have been done for testing and contact tracing?<br>• Are there any standardized protocols for testing of suspected and confirmed COVID-19 case?<br>• What is the procedure for contact tracing of confirmed case?<br>4. Have risk assessments been undertaken in respect of infection prevention and control activities at all levels of the healthcare system?<br>• Have referral pathways been defined?<br>• Have there been any changes in the provision of other services?
3. Information and communication system	1. What is the existing surveillance and management information systems at local levels?<br>• How are they being used?<br>2. What is the communication channel between the different levels of government?<br>• Are there any guidelines on reporting COVID-19 cases?<br>• How is the information being disseminated? (traditional, social media)<br>3. Have risk communication been undertaken in respect of timely data analysis and operational decision making?
4. Health Workforce	1. Are there adequate frontline health workers to handle the COVID-19 crisis? If not, why?<br>2. What trainings and preparedness activities are being undertaken with the workforce?<br>• Are all avenues to take advantage of remote training of staff by radio, digital media, etc. exploited?

	4. Can you tell us what motivates the health workers to work amidst the fear of COVID-19?<br>• Are plans being put in place to protect frontline workers?<br>• Do they comply with the need to avoid risky gatherings and transport?<br>• Is there any provision for motivation of health workers such as incentives, insurance etc?<br>• Can you tell us something about discrimination or stigma faced by frontline health workers during this COVID 19?<br>• In such situation, is there any provision of psychosocial support to them?<br><br>5. What is the role of health workers in preparedness and response activities?
5. Finance	1. Is there an estimate of additional funding needs for COVID-19 emergency preparedness and response measures?<br>• Is there any contingency fund, budget allocations, donor support, etc?<br>• How much of the needed additional funding has been mobilised and how much more is expected?
6. Supplies, logistics and infrastructures	1. How were the logistics and essential supplies managed?<br>• Do you think the current supplies and logistics are sufficient?<br>• Are timely activities being undertaken to build up stocks of drugs, consumables, and personal protective equipment (PPEs)?<br>• Are emergency procurement and distribution plans being developed (or in place)?<br><br>2. What has been done to strengthen the infrastructures of the health institutions for providing quality health services?<br>• Are adjustments being made to infrastructure (e.g.quarantine & isolation centers, expansion of laboratory)?
Recommendations & Way Forward	1. What are the limitations and challenges that you have experienced in managing COVID-19 cases?<br><br>2. What should be the way forward in terms of preparedness and response to COVID-19 or similar epidemic in future?<br><br>3. Would you like to express on any other issues that are left in our discussion?

4. Interview Guide (Stakeholders)

A Critical Analysis of Health System in Nepal; Perspectives based on COVID-19 response

Preamble:
Hello my name is Bihari Sharan Kuikel, a master's student in the Department of Public Health at Kathmandu University, Dhulikhel. This study seeks to critical analysis of Health System of Nepal Perspectives based on COVID-19

	**Questions**
	1. What is the role being played by different types of international agencies?<br><br>2. What coordination activities exist between the government and the international agencies in response operations?<br>• Technical guidance and support<br><br>3. How do the international agencies support to COVID-19 preparedness and response?<br>• Funding to Government<br>• Logistics supplies<br>• Infrastructure development<br>4. What lessons do we learn from this epidemic?<br><br>5. What should be the way forward in terms of preparedness and response to COVID-19 or similar epidemic in future?<br><br>6. Would you like to express on any other issues that are left in our discussion?
